# Como a Presença de Doenças Cardiovasculares pode Impactar na Morbimortalidade em Pacientes com COVID-19?

**DOI:** 10.36660/abc.20220225

**Published:** 2022-05-04

**Authors:** Alexandre de Matos Soeiro

**Affiliations:** 1 Hospital das Clínicas Faculdade de Medicina Universidade de São Paulo São Paulo SP Brasil Instituto do Coração do Hospital das Clínicas da Faculdade de Medicina da Universidade de São Paulo, São Paulo, SP – Brasil

**Keywords:** Doenças Cardiovasculares/complicações, Covid-19, SARS-CoV-2, Fatores de Risco, Doença Cardiopulmonar/mortalidade, Mortalidade Hospitalar/tendências, Troponina/efeitos adversos

Desde o início da pandemia, a doença causada pelo vírus SARS-CoV-2 denominada COVID-19, mostrou-se como uma afecção de espectro amplo e imprevisível, com pacientes apresentando-se praticamente assintomáticos, enquanto outros cursavam com comprometimento pulmonar grave, sendo essa a maior causa de morbidade e mortalidade agregada à doença.^1 - 3^

Já em momentos iniciais, a COVID-19 mostrou ter uma ligação com o sistema cardiovascular ampla e potencialmente alarmante. Os receptores da enzima conversora de angiotensina-2 mostraram ter uma interface direta com a patogênese viral, podendo ser a porta de entrada celular do mesmo nos pneumócitos tipo 2, macrófagos e cardiomiócitos.^1^ Sendo assim, pacientes com doenças cardiovasculares mostraram-se mais susceptíveis às formas graves da doença. Hipertensão, arritmias, miocardiopatias e doença arterial coronariana estavam entre as principais comorbidades presentes em pacientes críticos com COVID-19. Pacientes com doenças cardiovasculares (particularmente aqueles hipertensos) apresentam taxa de morbidade de até 10,5% após a infecção por COVID-19.^2^ No presente estudo, podemos observar uma relação semelhante. Ficou nítido nessa casuística brasileira como a presença de doença aterosclerótica e fatores de risco tradicionais isoladamente ou em conjunto foram capazes de impactar em mortalidade e prognóstico. Apesar do estudo apresentar uma casuística limitada, ele inclui somente pacientes de alto risco com índice de desfechos elevado, permitindo avaliar resultados de maneira consistente e seguindo exatamente a mesma linha da literatura internacional.^4^

Da mesma forma, a injúria miocárdica mostrou-se um potencial marcador de mortalidade na COVID-19. Mesmo após mais de dois anos da doença, os mecanismos propostos de lesão cardiovascular ainda não estão completamente estabelecidos, mas sugere-se que seriam dano direto aos cardiomiócitos, inflamação sistêmica, fibrose intersticial do miocárdio, resposta imune mediada ao interferon, resposta exagerada de citocinas por células T, disfunção endotelial, além de desestabilização da placa coronária e hipóxia.^1 - 3^

Elevações de troponina mostraram-se significativamente relacionadas à maior mortalidade e arritmias cardíacas. O aumento do marcador ocorre mais frequentemente em pessoas com doenças cardiovasculares crônicas do que em indivíduos previamente saudáveis. O aumento da atividade protrombótica, inflamatória e a hipóxia, contribuem para a ocorrência da injúria miocárdica. No entanto, presença de miocardite, cardiomiopatia induzida por estresse, insuficiência cardíaca aguda e lesão direta do cardiomiócito também contribuem para sua ocorrência. Até mesmo condições não diretamente relacionadas ao coração, mas comuns na COVID-19, levam ao aumento da troponina como embolia pulmonar, sepse e estado crítico do paciente.^5 , 6^ Também no estudo apresentado, a injúria miocárdica talvez seja o principal marcador prognóstico e o achado mais relevante nessa casuística. Observando os resultados, é possível constatar como a troponina impactou de maneira significativa e independente em mortalidade, mais do que qualquer outro escore, comorbidade ou fator de risco. Dessa forma, ela apresentou-se como o principal marcador prognóstico de maneira independente, predizendo inclusive elevada mortalidade em pacientes sem doenças cardiovasculares prévias ou fatores de risco acumulados.^4^

Em pacientes que necessitam de internação em unidades de terapia intensiva, a COVID-19 mostrou que a ocorrência de manifestações cardiovasculares é ainda maior. Arritmias cardíacas foram observadas em 16,7% dos pacientes internados, sendo presente em 7% dos pacientes que não necessitaram observação em terapia intensiva e 44% naqueles internados na UTI. Disfunções metabólicas, inflamação, ativação do sistema nervoso simpático seriam os principais fatores predisponentes das alterações de ritmo cardíaco.^2^ Tais achados são condizentes com o estudo apresentado, no qual a mortalidade dos pacientes considerados críticos chegou a 24% e o desfecho combinado de morte, ventilação mecânica e injúria miocárdica em 38% da população avaliada.^4^

Sendo assim, hoje temos um entendimento melhor do COVID-19 e suas manifestações cardiovasculares. Fica claro o quanto a presença de comorbidades cardiovasculares e as manifestações cardiológicas do COVID podem agravar o prognóstico, principalmente em pacientes críticos. A troponina se fixa cada vez mais como um dos maiores marcadores independentes de prognóstico da doença. Diversas lacunas sobre a fisiopatologia e o tratamento ainda permanecem sem esclarecimento, sendo alvos de futuros estudos clínicos.
