# Supplementary material for: Como a Presença de Doenças Cardiovasculares pode Impactar na Morbimortalidade em Pacientes com COVID-19?
Source: Arq Bras Cardiol. 2022 May 4;118(5):935–6. [Article in Portuguese] doi: 10.36660/abc.20220225 (PMC9368873; doi:10.36660/abc.20220225)
Supplement: Translation

# Translation: How Can the Presence of Cardiovascular Diseases Impact Morbidity and Mortality in Patients with COVID-19?

**Authors:** Alexandre de Matos Soeiro

**Affiliations:** 1 Brazil Instituto do Coração do Hospital das Clínicas da Faculdade de Medicina da Universidade de São Paulo, São Paulo, SP – Brazil

**Keywords:** Cardiovascular Diseases/complications, Covid-19, SARS-CoV-2, Risk Factors, Pulmonary Heart Disease/mortality, Hospital Mortality/trends, Troponin/adverse effects

Since the pandemic’s beginning, the disease caused by the SARS-CoV-2 virus, called COVID-19, has shown itself to be a wide-spectrum and unpredictable condition, with patients being practically asymptomatic. In contrast, others had severe pulmonary involvement, the major cause of morbidity and mortality associated with the disease.^1 - 3^

At an early stage, COVID-19 was shown to have a broad and potentially alarming link to the cardiovascular system. Angiotensin-2-converting enzyme receptors have been shown to directly interface with viral pathogenesis and maybe the cellular gateway for type 2 pneumocytes, macrophages and cardiomyocytes.^1^ Thus, patients with cardiovascular diseases were more susceptible to severe forms of the disease. Hypertension, arrhythmias, cardiomyopathies and coronary artery disease were among the main comorbidities in critically ill patients with COVID-19. Patients with cardiovascular diseases (particularly those with hypertension) have a morbidity rate of up to 10.5% after infection with COVID-19.^2^ In the present study, we can observe a similar relationship. It was clear in this Brazilian series how the presence of atherosclerotic disease and traditional risk factors alone or together were capable of impacting mortality and prognosis. Although the study has a limited series, it includes only high-risk patients with a high rate of outcomes, allowing the evaluation of results to be consistent and following exactly the same line as the international literature.^4^

Likewise, the myocardial injury proved to be a potential marker of mortality in COVID-19. Even after more than two years of illness, the proposed mechanisms of cardiovascular injury are not yet fully established. However, it is suggested that they would be direct damage to cardiomyocytes, systemic inflammation, myocardial interstitial fibrosis, interferon-mediated immune response, exaggerated

cytokine response by T cells, endothelial dysfunction, in addition to coronary plaque destabilization and hypoxia.^1 - 3^

Troponin elevations were significantly related to increased mortality and cardiac arrhythmias. Marker enhancement occurs more often in people with chronic cardiovascular disease than previously healthy individuals. The increase in prothrombotic and inflammatory activity and hypoxia contribute to myocardial injury. However, myocarditis, stress-induced cardiomyopathy, acute heart failure, and direct cardiomyocyte injury also contribute to its occurrence. Even conditions not directly related to the heart but common in COVID-19, such as pulmonary embolism, sepsis, and critical condition of the patient, lead to increased troponin.^5 , 6^ Also, in the study presented, the myocardial injury may be the main prognostic marker and the most relevant finding in this series. Looking at the results, it is possible to see how troponin significantly and independently impacted mortality more than any other score, comorbidity or risk factor. Thus, it presented itself as the main prognostic marker independently, even predicting high mortality in patients without previous cardiovascular diseases or accumulated risk factors.^4^

In patients who require admission to intensive care units, COVID-19 has shown that the occurrence of cardiovascular manifestations is even greater. Cardiac arrhythmias were observed in 16.7% of hospitalized patients, 7% of patients who did not require observation in intensive care and 44% of those admitted to the ICU. Metabolic dysfunctions, inflammation, and activation of the sympathetic nervous system would be the main predisposing factors for changes in heart rhythm.^2^ Such findings are consistent with the study presented, in which the mortality of patients considered critical reached 24%, and the combined outcome of death, mechanical ventilation and myocardial injury in 38% of the population evaluated.^4^

We now have a better understanding of COVID-19 and its cardiovascular manifestations. It is clear how much cardiovascular comorbidities and the cardiological manifestations of COVID can worsen the prognosis, especially in critically ill patients. Troponin is increasingly established as one of the major independent prognostic markers of the disease. Several gaps in pathophysiology and treatment remain unclarified, being targets of future clinical studies.
